# Two heads are better than one: current landscape of integrating QSP and machine learning: An ISoP QSP SIG white paper by the working group on the integration of quantitative systems pharmacology and machine learning

**DOI:** 10.1007/s10928-022-09805-z

**Published:** 2022-02-01

**Authors:** Tongli Zhang, Ioannis P. Androulakis, Peter Bonate, Limei Cheng, Tomáš Helikar, Jaimit Parikh, Christopher Rackauckas, Kalyanasundaram Subramanian, Carolyn R. Cho, on behalf of the Working Group, Ioannis P. Androulakis, Peter Bonate, Ivan Borisov, Gordon Broderick, Limei Cheng, Valeriu Damian, Rafael Dariolli, Oleg Demin, Nicholas Ellinwood, Dirk Fey, Abhishek Gulati, Tomas Helikar, Eric Jordie, Cynthia Musante, Jaimit Parikh, Christopher Rackauckas, Julio Saez-Rodriguez, Eric Sobie, Kalyanasundaram Subramanian, Carolyn R. Cho

**Affiliations:** 1 University of Cincinnati, Cincinnati, OH 45267 USA; 2 Rutgers University, New Brunswick, NJ USA; 3 Astellas Pharma Inc, Northbrook, IL USA; 4 Bristol Myers Squibb, Princeton, NJ USA; 5 Department of Biochemistry, University of Nebraska-Lincoln, Lincoln, NE USA; 6 GSK, Collegeville, PA USA; 7 Pumas-AI, Baltimore, MD USA; 8 Department of Mathematics, Massachusetts Institute of Technology, Boston, MA USA; 9 Applied BioMath, Concord, MA USA; 10 Merck & Co., Inc, Kenilworth, NJ USA

**Keywords:** QSP, Machine learning, Review, Commentary

## Abstract

Quantitative systems pharmacology (QSP) modeling is applied to address essential questions in drug development, such as the mechanism of action of a therapeutic agent and the progression of disease. Meanwhile, machine learning (ML) approaches also contribute to answering these questions via the analysis of multi-layer ‘omics’ data such as gene expression, proteomics, metabolomics, and high-throughput imaging. Furthermore, ML approaches can also be applied to aspects of QSP modeling. Both approaches are powerful tools and there is considerable interest in integrating QSP modeling and ML. So far, a few successful implementations have been carried out from which we have learned about how each approach can overcome unique limitations of the other. The QSP + ML working group of the International Society of Pharmacometrics QSP Special Interest Group was convened in September, 2019 to identify and begin realizing new opportunities in QSP and ML integration. The working group, which comprises 21 members representing 18 academic and industry organizations, has identified four categories of current research activity which will be described herein together with case studies of applications to drug development decision making. The working group also concluded that the integration of QSP and ML is still in its early stages of moving from evaluating available technical tools to building case studies. This paper reports on this fast-moving field and serves as a foundation for future codification of best practices.

## Rationale

Predictive mathematical modeling has become an established element of drug discovery and development due to the totality of its impact on individual programs predicting, for example, preclinical-clinical translation, therapeutic index, optimal dosing, and drug-drug interactions, as well as reducing the size and number of clinical trials [1, 2]. Awareness of the need for modeling is being driven in parallel with the establishment of high quality, high dimensional preclinical and clinical data warehouses [3–5]. Approaches to predictive modeling have been developing and recently receiving attention from two major directions: quantitative systems pharmacology (QSP) which describes hypothesized or assumed mechanistic relationships in a mathematical formalism, and machine learning (ML), which applies unbiased algorithms to explore correlations in experimental data. The question is whether these two apparently disparate approaches may be integrated, and what value may arise from such integration (QSP + ML).

This White Paper describes the current achievements and possible future directions of early QSP + ML work from the perspective of the working group on QSP + ML within the ISoP QSP Special Interest Group (SIG). Membership of the working group reflects the diversity of backgrounds and expertise in the QSP community, from academic research in mathematical biology and chemical engineering to industry pharmacometrics applications. Discussions of members’ research have covered a broad range of methods and applications that are represented here. Integrating QSP + ML also spans a broad range of research objectives from increasing physiological understanding and predictive power to reducing the computational burden and complexity of analyzing large QSP models.

We begin with a brief review of the considerations of QSP and ML separately. Considerations for integrated QSP + ML are organized into four categories of current activity in the field, followed by illustrating case studies. We conclude with operational concerns of implementing ML within an existing pharmacometrics research groups and end with a future perspective. A glossary of common ML terms is provided as a quick reference. More specialized ML methods, not in common use, are included in the overview of current methods with references as a guide for interested readers.

## Background

### QSP modeling supports all stages of the drug development pipeline

The term QSP was coined in 2011 in an NIH white paper as an intersection of mathematical modeling and experimental approaches focused on drug pharmacology [6]. QSP-based predictive modeling using QSP has successfully supported many facets of drug development, including regulatory decisions [7–10], setting pre-specified goals for program go/no-go decision points, characterizing physiological / therapeutic mechanisms of action for single or combinatorial approaches, treatment optimization, and response to proposed dosing regimens [11, 12]. Agharmiri et al. [13] provides a comprehensive overview of QSP models, their application, and growth across different disease areas over the last three years (2018 to 2021).

The QSP modeling approaches apply existing knowledge of dynamical and nonlinear molecular mechanisms as a theoretical framework to test our understanding, contextualize new data and predict the outcome of intervention. Several mathematical and computational approaches have been used to encode QSP models, including ordinary and partial differential equations (ODE, PDE), logic-based methods, and constraint-based approaches [14]. It is the representation of existing knowledge – the crafting of assumptions – that presents the key challenges of QSP modeling: the selection of key molecular drivers, the generalization of mechanisms specific to physiological context, disease and human sub-populations [15] and the assignment or derivation of (often unobserved) parameters with the associated “curse of dimensionality” requiring more data to cover the parameter space. Furthermore, QSP modeling is labor-intensive. Model building is still largely performed by manual distillation of a large volume of scientific literature, often by one individual. Characterizing the model (establishing accuracy, sensitivity, reducibility, reproducibility) also requires manual distillation of available data and moreover, creates a substantial computational burden since QSP models typically comprise dozens or more dynamical variables and even more parameters.

### Machine learning allows data driven analysis as well as dimension reduction

Analyses of high dimensional data commonly leverage ML approaches such as classification, regression, clustering, associated rule learning, image processing, and ranking (for a primer on ML for life scientists see [16]). The ability to simultaneously observe 1000’s to 10,000’s of properties of a system across multi-layer data (genes, proteins, metabolites, etc.) does not require an assumption of key drivers: all possible molecular players are analyzed. Furthermore, inference or discovery of unknown associations among observables is possible. Inference has provided functional annotation for unknown nucleic acid sequences and characterized networks of associated functions among observables. However, the identification of testable mechanistic hypotheses has been widely recognized as one of the most significant challenges due to the general “black-box” nature of ML approaches [17]. The strength of QSP modeling to address this key weakness of ML, and the strength of data-driven ML to address the QSP weakness of manually building assumptions, suggests that integrated QSP + ML approaches offer the best of both.

It should be noted that the harmonization of data is one of the most important considerations in the analysis of high dimensional and/or integrated data. Preprocessing and cleaning of data includes imputation of missing data, normalization, handling of categorical variables, and the detection and handling of multicollinearity and systematic bias/error. Data harmonization is a central consideration of ML approaches but is also important in QSP models which often rely on integrating data from multiple sources.

Another strength of ML is to help reduce the manual labor, complexity, and execution of QSP modeling and simulation. Because comprehensive QSP models can be computationally expensive to solve and to characterize the multi-dimensional parameter space, surrogate ML models can be initially developed by training them with sample input–output combinations from QSP models, and subsequently used for further predictions. Surrogate ML models (or metamodels) have been used in engineering and physics to scale up simulations of multiscale models, providing opportunities for the life sciences field to adapt some of the methods [18].

## Current approaches

The working group has identified four categories of application for integrated QSP + ML approaches.

1. Parameter estimation and extraction. Inferring parameter values for defined QSP models and reconciliation of model behavior with published qualitative and quantitative data
2. Model Structure. Inferring relationships including logic networks of large QSP models from a variety of data types. Related to this are methods enabling the evaluation of sensitivity and uncertainty of parameters and model structures including constraint-based approaches. These methods also are applied to extracting conclusions from heterogeneous populations of QSP models.
3. Dimension reduction. Methods to extract variables from high dimensional data, whose behavior most informs outcome.
4. Stochasticity and virtual populations. The assessment of stochastic considerations such as predicting the impact of genetic variants and mechanistic sources of variability

### Parameter estimation and extraction

Parameter estimation methods for large scale systems of differential equations are, from a numerical analysis perspective, problems of minimization or maximization of a defined cost function. In general, most ML problems reduce to optimization problems. Parameter estimation methods have been reviewed extensively (see for e.g. [19]). For QSP models, the estimation is chronically under-determined due to limited clinical and preclinical pharmacology data [20]. Successful approaches have begun with a characterization of parameter space [14] using, for example, virtual populations [21, 22] to find parameter sets that generate outputs to be consistent with observed clinical data. Additional virtual population considerations are needed for strongly nonlinear models [23].

Successful implementation of ML for the direct analysis of pharmacometrics data hinges on the robustness of datasets for training and testing that capture the distribution of intrinsic and extrinsic factors of interest. Clinical trial data may not be suitable if the trial is small or has missing or irregular data. Furthermore, there may be no clear mechanistic association from clinical events to individual patient characteristics and/or QSP model parameters. ML has been applied to identify such associations as described in the thrombosis prediction case study, below [24]. In this example, logistic regression was used to generate the probability of a clinical event for each virtual patient simulated from the QSP model. Other methods, such as gradient boosted decision trees, deep neural networks and multitask deep learning (MDL) may also be used. MDL or transfer learning can simultaneously use features or biomarkers as input data and predict multiple clinical events or outputs. This allows the use of a large dataset to improve the prediction accuracy of small data sets and has been used, for example, to classify biological phenotypes from images [25].

Semi-automated extraction of parameters from the literature has also been accomplished using natural language processing, however additional analysis is required to ensure the extracted data are applicable in a particular QSP setting. Together with a ML model checking framework [26], this approach has been applied to a combined of parameter selection and QSP model selection to model immune dysregulation in children prone to a specific viral infection [27].

### Model structure

ML methods to identify QSP model structure in a data-driven manner rather than a manual digest of prior knowledge is an active area of method development. Application to drug discovery and development is underway with some published exploratory case studies, however these methods have not yet been widely assessed or validated.

The ML methods that have generated the most case studies have focused on identifying the regulatory mechanism logic of gene expression, signaling pathways and cell fate. These methods pragmatically focus on semi-quantitative data that are typically used to investigate biological decision pathways and have developed alongside refinements in the generation of experimental data. As experimental perturbations can improve our understanding of regulatory pathways [28], rich mutliplexed data are being coupled with ML-based model structure generation to identify therapeutic approaches, for example, to control cell fate [29] and to identify personalized cancer therapy [30]. These approaches offer the opportunity to build a QSP model supporting the full pipeline of activities starting with target identification, validation, and model refinement as questions become more focused later in clinical development.

**Fig. 1:**
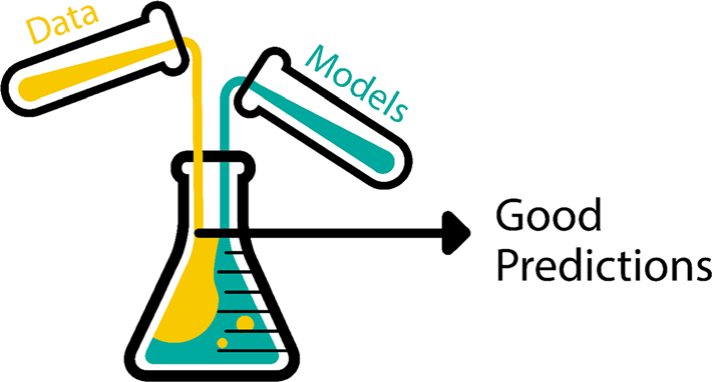
Scientific machine learning is model-based data-efficient machine learning. How do we simultaneously use both sources of knowledge? While lack of prior knowledge of mechanism can be supplemented by machine learning on data, scientific machine learning methods show that machine learning on small data can be supplemented by encoding mechanistic principles into the machine learning architectures. Thus, the important factor for achieving good predictive power is the total combination of data and mechanistic information encoded into these hybrid models

Traditional “black box” deep learning approaches like convolutional and recurrent deep neural networks eschew prior information for neural network flexibility. In comparison, mechanistic models such as QSP models routinely incorporate prior mechanistic understanding, such as the prior models themselves as the structural prior to reduce the data requirements (Fig. 1). The practice is gaining new momentum when combining with the modern computational power in the research field of scientific machine learning (SciML) (also sometimes referred to as physics-informed machine learning or science-guided machine learning) and uses several different approaches to incorporate known mechanism into machine learning architectures and training processes.

One branch of SciML focuses on Physics-Informed Neural Networks (PINNs) [31] which has been applied in systems biology to generate Biologically Informed Neural Networks [32]. In this approach, mechanistic regularization is added to the neural network by modeling the neural network NN(t) as the solution to the QSP model represented by an ODE ${u}^{^{\prime}}=f(u,t)$. These techniques have been shown to perform well on sparse data by using the model form to compensate for the unknown quantities. In this formulation, parameter estimation can be performed simultaneously to the neural network training process, making it potentially a tool for identification with small data. In addition, one can choose neural architectures which impose constraints that must be satisfied in the evolution of the system. In fluid dynamics, this has been shown to improve data efficiency even further [33]. However, this method is very computationally expensive. A comparison of standard ODE solver based parameter estimation with the DeepXDE package [34] used in Biologically-Informed Neural Networks showed local optimization of parameters was 100 × to 10,000 × more expensive than traditional approaches [35].

Another SciML approach is “grey box” modeling using the neural network as a universal approximator to represent unknown portions of models. This is the approach taken in the universal differential equation framework [36], also referred to as hybrid models or grey-box models with universal approximators. Universal ODEs can be simplified to the form ${u}^{^{\prime}}=f(u,NN\left(u\right),t)$. For example, a one-compartment pharmacokinetics model with unknown nonlinear feedback can be expressed in the form:

$$\left[ {Depot} \right]^{^{\prime}} = - Ka NN\left( {\left[ {\left[ {Depot} \right],\left[ {Central} \right]} \right]} \right)$$

$$\left[ {Central} \right]^{^{\prime}} = Ka NN\left( {\left[ {\left[ {Depot} \right],\left[ {Central} \right]} \right]} \right) - \frac{CL}{V}\left[ {Central} \right]$$

where the final activation of the neural network could be chosen to impose positivity of its output. Such a form can then be used to extend full mechanistic models to find terms missing from the original description via symbolic regression. This approach has been demonstrated in other fields such as battery engineering [37] and climate modeling [38] increasing the prediction accuracy over state-of-the-art mechanistic models with only mild data requirements. This approach has also been extended to Bayesian probabilistic forms [39] for calculating the probability of missing or unknown mechanisms. Universal differential equations do require specialized numerical differential equation solver implementations like DiffEqFlux in order to be accurately and efficiently trained.

Recent ML research has focused on addressing computational challenges specific to QSP modeling such as stiffness. Techniques like PINNs can be susceptible to training failures on highly stiff models [40] potentially due to neural networks having a low frequency bias [41]. New architectures [42, 43] have been developed for stiff biophysical models where approaches such as PINNs, recurrent neural networks, long short-term memory networks, and convolutional neural networks can fail due to an ill-conditioned optimization process. More specialized architectures will likely be required to reach the accuracy and robustness for ML applications in QSP.

### Dimension reduction

Since the aim of a QSP model is putatively to understand molecular interactions at the site of action and their impact on the overall physiology of long-term disease progression in general and different sub-populations, it is expected that QSP models operate on multiple time and length scales i.e. in a high dimensional space. In order to model these complex systems, dimension reduction methods are often used [44, 45]. These methods identify a subset of variables and parameters that describe the mechanisms of interest, optimally balancing computational performance and complexity [46, 47]. Dimension reduction is helps with interpretability of a model, since an overly complex model may obscure a decision maker’s ability to establish interpretable hypotheses. Thus, trade-offs between performance and complexity need to be balanced [46, 48].

**Fig. 2:**
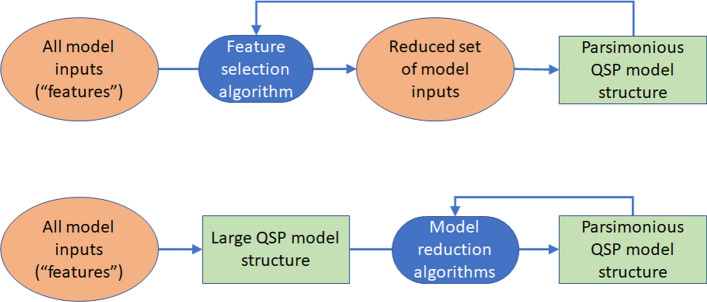
Procedure for integrating ML and QSP modeling in two different ways. Top: ML algorithms could be used to select features, which then could be used to develop QSP models that include only the highly relevant features; Bottom: alternatively, comprehensive QSP models that include most features could be first developed, then ML algorithms and sensitivity analysis could be used to reduce the scale of QSP model until smaller, more focused QSP models are achieved

Indeed, feature selection (FS) can assist in the identification of a maximally informative subset of variables that capture the essential behavior of a system. FS is a central problem of ML, where a minimal subset of inputs (features) is selected according to a defined criterion (e.g. a subset of genes whose expression predicts response to a drug treatment. FS methods are data-driven and can inform QSP model structure by identifying the minimal physiologically meaningful representation to enable mechanistic interpretation and prediction [49]. The higher efficiency achieved using FS is clear when comparing a FS + QSP strategy with a more traditional QSP model dimension reduction strategy (Fig. 2).

- FS + QSP. Starting with all measured features, FS is performed to identify the subset for QSP modeling. The reduced model is parameterized and checked, and feature selection is re-evaluated until an optimal structure is identified.
- QSP only. A QSP model is built using the complete feature set, parameterized, checked, and dimension reduction techniques are applied. As required, dimension reduction, parameterization and/or checking are re-evaluated until an optimal model structure is identified.

This simplified strategy of FS + QSP considers an independent FS method. However, it is difficult to decouple FS from other ML approaches that may also be applied to a specific question or context. It is thus difficult to propose universal and ubiquitous methods for FS and dimension reduction. Comprehensive analyses [50] have helped drive a consensus that univariate FS methods treating variables independently and one-at-a-time are suboptimal and should be applied in consideration of the ML task. Thus, the three broad categories of FS algorithms – the filter, wrapper and embedded approaches – include this consideration [51, 52]. Filter methods resemble an unsupervised approach, where FS is performed independently of ML. Wrapper methods resemble more supervised methods in the sense that feature selection is validated based on the performance of the subset to specific ML tasks. Embedded methods perform the ML and FS tasks simultaneously by incorporating (embedding) the FS within the learning algorithm. Embedded methods usually incorporate FS in the form of a set of constraints in an overall multi-level optimization problem which attempts to maximize the ML task while simultaneously minimizing (some metric of) model complexity [53–55].

FS algorithms can be further classified as: (a) methods that preserve the nature and meaning of the features resulting in a “reduced” dimensionality representation composed of a true subset of the original feature set; and (b) transformation methods whereby “new” features are created through manipulations; i.e. linear or non-linear transformations of the original features [56]. Each class has its own advantages and disadvantages, with the most obvious being that purely reduction methods preserve the nature, character, and physical meaning of the features and as such model development and/or interpretation comes more naturally. On the other hand, transformation methods can achieve substantial dimensionality reduction – since the variables are replaced with complex transformations of the original features. However, the interpretation of the transform variables becomes quite challenging: transformed variables become (non)linear functions, or projection on (non)linear spaces of the original variables and have, therefore, lost their physical meaning. Interpretation becomes important as we move towards the development of, so-called, *digital biomarkers* able to predict [57].

The search for quantitative structure–activity relationships (QSAR) is one of the fields that have most benefited from combined developments in ML and FS. QSAR brings together a multitude of interesting problems: (1) a rich and non-uniquely defined input space, since often molecular features are computationally defined expressing a multitude of structural characteristics and molecular descriptors; (2) a formidable ML problem since the relations between structure and activity need to be inferred; and (3) by its nature the feature space can easily become very high-dimensional, thus necessitating the reduction of its dimensions [50, 52, 58].

### Stochasticity and virtual populations

There are additional case studies of ML applications in pharmacometrics that do not yet create their own category [59–61], including the use of stochastic approaches to predict the effect of random genetic evolution or small populations [62, 63]. However, stochastic approaches are more widely used for the generation of virtual patient populations.

Achieving increased confidence in QSP model predictions for critical decision-making requires model calibration, parameter estimation, sensitivity analysis, uncertainty quantification, and generation of virtual patient populations. Related to their use in parameter estimation, virtual patient populations are used to simulate how variability in patient physiological characteristics explains mechanistic contributions to the variability in response to drugs or other clinical outcomes. The use of virtual population modeling has gained significant attraction over the last decade [21, 64–66]. Traditional methods employ initial sampling of a subset of model parameters to construct a large set of potential virtual patient candidates followed by a filtering/rejection step based on different constraints to generate the final population. The method ensures that the final population of models comprise physiologically plausible models constrained by the feature ranges in the observation. ML methods such as prevalence weighting and other heuristic methods that use Markov chain Monte Carlo sampling have been used to construct virtual population that match the proposed data density [21, 22, 64, 67, 68].

The inverse problem of inferring parameters of the mechanistic model has often been formulated as a Bayesian inference problem. Novel generative ML models, such as flow and generative adversarial network normalization, are increasingly investigated for parameter inference of mechanistic models and virtual population constructions [69–71]. Normalizing flow-based methods are currently used to infer stochastic model parameters in cases where experimental data are acquired from a single individual [71, 72], but they can be readily extended to construct virtual population QSP models. Novel generative adversarial network (GAN) configurations have been shown to allow for construction of populations deterministic models [70] by addressing complex model parameter inference scenarios involving data from heterogeneous populations.

Sensitivity analysis, uncertainty quantification and virtual population generation requires performing hundreds of thousands of model simulations, another example of the need for ML-facilitated dimension reduction.

## Case studies

The utility of the integrated QSP + ML approach is, of course, to support decision making during drug discovery, development and registration. Application of a QSP + ML approach requires a determination of the key decision to be addressed, how the model will inform the decision, and how quickly the decision is needed. This will, in turn, determine the balance between pragmatism and deep mechanistic understanding that is required. A number of case studies are reviewed, illustrating current impact of the QSP + ML method.

### Prediction of therapeutic window for thrombosis treatment

QSP modeling in thrombosis can shed light on important aspects of hemostasis and thrombosis [73, 74]. Mechanistic models of the coagulation pathway, and more generally thrombosis, have been used extensively to characterize the kinetics of coagulation and clot formation [75]. Linking the mechanistic outputs of such a model to the clinical endpoints that are reflective of the benefit and risk balance of anti-coagulant therapy should be possible. However, there is uncertainty regarding the mechanistic relationship between clot formation and venous thromboembolic events and bleeding. Because of this uncertainty, machine learning was used to help quantify those relationships [73, 74].

The suitability of clinical data for ML can be limited. QSP models can generate data to represent population uncertainty, such as age, gender, pre-existing conditions, missing dose, but still give the output in a uniform manner, which is more suitable for ML. Associations between simulated patient characteristics and multiple trial events may provide insight due to the effects of undescribed biological and physiological mechanisms. Real world event rate data reported from multiple trials using different drugs at different doses was used to tune an event prediction algorithm.

The application of this QSP and ML approach supported clinical development of anti-coagulants, including comparing them to competitor molecules and standards of care, informing the design of the venous thromboembolism (VTE) prevention trials in orthopedic surgery, comparing the efficacy and safety of the lead vs. backup, evaluating potential combination therapies, and predicting dosing and therapeutic window for VTE treatment and prevention.

### Prediction of drug induced liver injury from QSP and gene expression data

Although empirical PK/PD is commonly applied to model toxicity, QSP modeling of injury at the cellular and tissue levels – quantitative systems toxicology (QST) – offers advantages for species translation and understanding intraindividual differences that could improve toxicity prediction [76]. The integration of QST with omics data is the subject of the TransQST consortium [77]. An early case study of the approach coupled a QSP model of liver homeostasis with in vitro data to predict in vivo toxicity [78]. A general theoretical framework to generate QSP models from curated networks and expression data was proposed by Kulkarni et al. by focusing on gene regulatory networks alone [79]. Following this approach, interactions between genes and known hepatoxicity mechanisms were identified from the literature using natural language processing and used to expand a prebuilt QSP model [80]. The QSP model described the mechanisms of necrosis, steatosis and cholestasis comprising 112 coupled differential and algebraic equations were constructed, including fat, antioxidant and bile metabolism and transport. Toxicogenomic data was then generated for a specific drug of interest, and ML used to find the differentially expressed genes. The gene list was overlaid on the expanded ML + QSP network to convert gene-level changes into hypothetical perturbations of the ML + QSP homeostasis. Simulations were run to understand the relative impact of multiple mechanistic perturbations, and to predict hepatotoxicity.

### QSP model structure inference and reduction of high dimensional data

#### Reduction of high dimensional data: Boolean networks and circadian pharmaco-pathomics

Boolean networks model the binary on/off behavior of the variables (elements of the network) and infer the simplest structural relationships that describe the overall behavior of the system, most often applied to describing transcriptomic network behavior. Recent work developed methods for the identification of causal relationships within high dimensional data and for complex dynamic behavior such as circadian rhythms [81].

#### Regulatory network prediction of T cell differentiation

The immune system has been modeled by a variety of mathematical approaches, including ODE-based models [82]. The data generated to investigate mechanisms of immune cell activation are predominantly semi-quantitative, with the important aspects being the presence or absence of signaling molecules such as cytokines and the identity and activation state of immune cells measured, for example, by the expression of cell surface markers such as CD4, CD8, CD28 etc. The concentrations of cytokines over time, frequency of cell counts, and other quantitative measures over time may also be of interest but are more difficult to determine.

Modeling the regulatory decision pathways of cell activation using a logic-based approach is a natural way to represent available data [83] and allows the prediction of T cell fate. In particular, proper activation and differentiation into specialized effector T cells and inducible regulatory T cells are essential for orchestrating the balance between protective immunity and undesired inflammation suppression. Plasticity, the ability to change phenotype and acquire mixed or alternative fates, is a critical property of T cells, enabling them to adapt their function and response to changing environments and contexts. Extracellular cues regulate T cell plasticity via complex signaling, metabolic, and epigenetic networks. The ability to design T cell microenvironments that can elicit specific programming regimes has translational potential for many diseases (e.g., cancer, autoimmune diseases, and transplantation). To understand better how extracellular cytokine milieu and signaling drive T cell differentiation, a logical model of signal transduction networks has been used to comprehensively interrogate its dynamics under hundreds of environmental conditions. ML-based classification of the dynamic response resulted in new evidence that T cell fates depend on specific combinations of stimulating cytokines and quantitative (dosage) and temporal (timing) dynamics [84, 85] and discovery and characterization of novel complex (multi-fate) T cell phenotypes [84, 86] as well the extracellular “recipes” that can potentially regulate the balance of each phenotype [84].

#### Reduction of high dimensional data: network inference

Vaccine hyporesponse in the elderly is associated with chronic inflammation and has been studied using multi-layer molecular profiling in order to identify mechanisms to target for therapeutic discovery [87]. In order to design a new study to meta-genomic profiling of microbiome, a non-human primate (NHP) study was performed to understand how changes in host immune system response to vaccination (or lack of response) was associated with changes in microbiome in old versus young animals [88]. The purpose of these studies was to characterize the behavior of molecular entities that may play a role in diminished vaccine response in older adults as targets for vaccine adjuvant discovery. The ML-based analyses reduced a very large set of data to a small, interpretable set of interactions to support adjuvant identification, and to be developed into a QSP model to support adjuvant validation and the putative clinical development program.

A novel machine learning method was developed with the above considerations [48]. The method identifies the subset of entities (e.g. genes, proteins, metabolites, cell types) that is the most useful for predicting the behavior of the whole system. It provides important improvements on similar methods, eliminating the need for the user to adjust machine learning parameters that is typical for such methods, and producing a sparse, parsimonious network. Importantly, the method is independent of the distribution of the data, allowing the integration of disparate data types – transcriptomic, proteomic, metabolomic, cell profiling, and demographic data from each subject. Both human and NHP vaccine hyporesponse data [87, 88] were analyzed and the network of entities in common between datasets was identified. Functional annotation [89] and visualization was performed manually in collaboration with bench scientists to interpret how sub-networks were connected. From this process, the structure of interlinked pro-inflammatory pathways including IL-6, Il-23, monocyte and dendritic cell activation, TNF-alpha and T-cell differentiation were identified to influence B cell class switching and overall response. The relationships between microbial metabolites and dendritic cell maturation as well as B cell antibody production were proposed [90]. These hypotheses are being tested using data from an on-going study.

## Conclusions, discussion and future perspective

### Practical considerations: Implementing ML in a drug discovery & development setting

The adoption of new technologies generally follows the same lifecycle. At first, there will be a group of innovators and early adopters, which eventually leads to a majority group of users (both early and late stage), followed by a group of late-comers [91]. At the same time, the technology itself has a lifecycle as it changes from a new technology to a growth technology to a mature technology to a declining technology. ML is an interesting case because it has been around for decades; it is really only recently, with the advent of new hardware and algorithm advances, that ML has seen increased adoption in science and society. ML overall is probably in the mature stage of development, but in the growth use phase within society. Within the pharmaceutical industry, the use of ML is not as advanced as other industries, and may still be in the early adopter stage, as companies are starting to identify applications for ML in both the commercial and development space. Whether ML succeeds and becomes the transformative technology in the pharmaceutical industry remains to be seen.

**Fig. 3:**
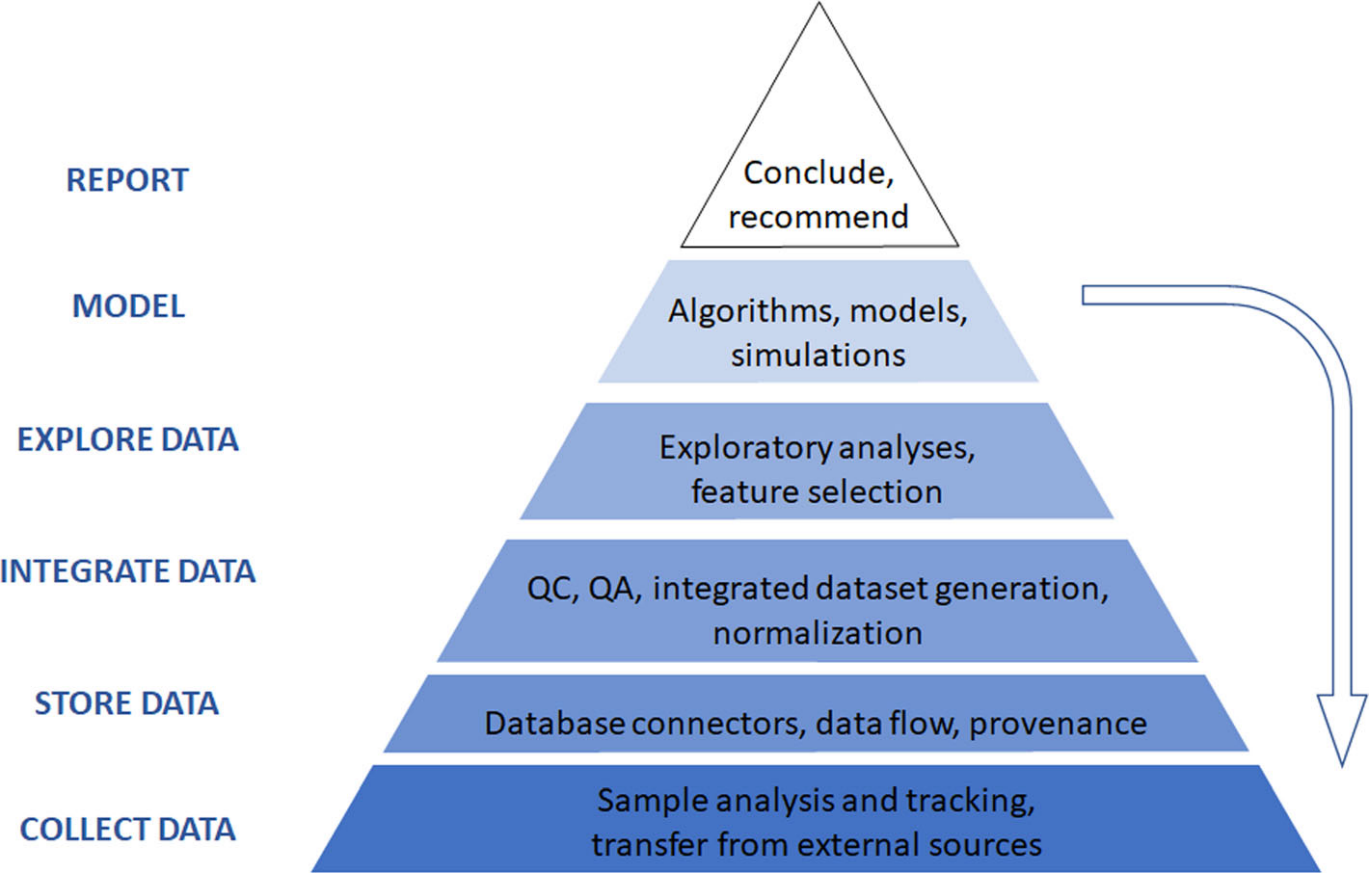
QSP + ML Hierarchy of Needs. Based on Maslow’s Hierarchy of Needs, companies must satisfy lower levels before moving to higher levels. Additionally, QSP models establish the framework for identifying the most informative data for scientific discovery, requiring an iterative workflow to generate new data

Henstock [92] argues that implementing ML within a company follows a hierarchical approach, which he refers to as the “AI hierarchy of needs”. Based on Maslow’s hierarchy of needs for personal growth, lower levels of the hierarchy must first be satisfied before moving upwards to the next level (Fig. 3). At the very bottom of the hierarchy, there must be data, algorithms, and hardware before one can even think about using ML. Then the company needs to be aware that ML can be used to solve the problem. Once aware, companies realize they do not have the expertise themselves to solve it, so they must contract or partner with others that can. Over time, the company starts to build the resources to do ML internally and eventually starts to do so. With continued effort these capabilities mature, and the company becomes reliant on ML, capable of handling most problems with their own internal solutions, before maturing into a full-fledge AI-driven organization, where the company derives its competitive advantage from its ML algorithms. Most Pharma companies are somewhere between the AI Outsourcing and Collaboration stage and ML-capable stage. No pharma company is AI-driven, and one could even argue that this may be impossible for a Pharma company; that Pharma should target an AI-enabled organization where AI is just one factor used to derive its competitive advantage.

To start to implement ML within a company requires the obvious resources like qualified personnel and computer hardware/software able to process big data. Further, there are cultural constructs that can improve the adoption of ML at a company [93]. Below are a few constructs identified from experience to improve the success of ML at a company.

ML can be oversold as a magic solution to every problem, but it is not. The lay press has given the impression that ML will one day rule the world (as in science fiction movies). However, ML does have its limits despite the great strides in the use of ML from self-driving cars to improving health care that have demonstrated the vast potential of the approach. The right framework to think through is how to better understand an organization’s decisions, how those decisions are currently made, when are they going to be made in the future, what information would be helpful to have at the time of the decision being made, and then, finally, how the data can be collected, processed, analyzed, and translated into an insight to inform that decision (Ryan Moore, personal communication, 2021). Sell the vision of what ML looks like when effectively integrated into this framework.

When first starting a ML group, don’t let the first problem you tackle be a “moon shot”. Don’t start with a big problem. Start small. Look for quick wins and early successes. First impressions matter. Tackling a hard problem and producing a less than satisfactory result will tarnish the perception of all future work, particularly when other projects fall short. Start with a smaller problem and compare results to traditional methods. For example, a supervised classification problem could compare a neural network to results from a logistic regression analysis. Once the group has some successes under their belt, start to tackle bigger, more ambitious projects, and then move onto the “moon shots”.

Collaboration is mission-critical. Often the data scientists performing the ML do not have the same skill set as the subject matter experts of the problem at hand, an example being using ML to classify the presence or absence of tumors in radiological scans (few radiologists can program use ML). Whatever projects are started, they should be done in a collaborative, inter-disciplinary manner. Data scientists should never work in a silo and then present their results to teams after the analysis is complete. Working in a collaboration leads to “buy-in” from all team members and a sense of ownership in the results, which may lead to greater use adoption in the future. Collaboratory teams can also lead to synergies, or identify project bounds, that a single data scientist may not be aware of. At the same time, the data scientist should be aware of turf issues among groups. For example, there has always been an uneasiness between ML and statistics (and the age-old question – “what is the difference between ML and statistics anyway?), which may translate to how these groups work together. Going back to the use of logistic regression for classification, traditional statistics groups may see that as their purview and may not be happy with another group doing it, so it’s important to be aware and delicate in this regard.

Communication is the key to success and there are many facets to this. First, there must be clear communication and agreement on the problem to solve. Albert Einstein once said that “If I were given one hour to save the planet, I would spend 59 min defining the problem and one minute resolving it.” A great solution is worthless if it solves the wrong problem. Second, the importance of hiring data scientists that can explain what it is they are doing cannot be overstated. Invariably, ML presentations to non-ML scientists require at least some level of explanation regarding the methods being used. Data scientists that cannot explain their methods in everyday language lower the chance for a particular model to be accepted and adopted. Some companies have taken to hire model translators, who are not involved in the actual modeling itself, whose job is to translate the problem to the data scientists and then to help the data scientists explain the mechanics and modeling results into common language that everyone can understand it.

### Challenges and future perspectives

The current state of the art does not include fully integrated hybrid QSP + ML models. The case studies presented are ML-assisted QSP modeling, using ML to address weaknesses of QSP models. ML can be applied to parameterize QSP models, analyze QSP model simulation, optimize computational burden and one-time feature selection to inform QSP model structure. The desired future state is to use hybrid models to iteratively uncover black box mechanisms through rigorous, systematic analysis, linking therapeutic interventions to the probability of clinical events.

From our perspective, the success of developing new drug therapies will be increased if QSP modeling is applied earlier and deeper. For example, even before data is collected, QSP models serve as a framework for a research team to align on assumptions, to prioritize the key gaps where data should be collected, and to design experiments that maximize the value of the investment into new data generation. Integrating ML into this process enhances all aspects of this process.

A continuing challenge is the lack of high-quality, high-volume clinical data. Advances in the technologies supporting decentralized trials such as smart phones, wearable and blood self-collection devices, and in the establishment of collaborative clinical data warehouses, offer new data resources but are accompanied by new challenges in bridging and integrating data. ML is used to simulate QSP models, generating virtual patients that reflect variability of model parameters. This approach is used to predict distribution of response given, for example, genotypic characteristics of a population. Hybrid QSP + ML models improve this surrogate model approach by concurrently optimizing model structure with simulation, rather than building the simulations from a fixed QSP model.

The next generation of QSP modeler will be called upon to cross yet more interdisciplinary boundaries. The successful impact of QSP is entirely due to the mathematical modelers who are also disease biologists and clinical pharmacologists, delivering analyses. These modelers successfully addressed key questions during the development process, through regulatory agency submissions, that could not otherwise be answered. The expertise of numerical analysts is now needed to build the collaborative expertise necessary for identifying and addressing a new class of questions. ML expertise conversely promises tools for automating the modeling process and providing accessibility for non-modelers and modelers, alike (does one have to be an auto mechanic to drive a car?).

The integration of QSP and ML is in its early stages of moving from evaluating available technical tools to building case studies. Such integration offers multiple advantages from providing data-driven QSP model parameterization, to imposing a QSP model framework to increase interpretability of high dimensional data and fully data-driven QSP model structure discovery.

Driven by advances in data acquisition and warehousing technology, as well as the improved understanding of key questions where QSP + ML can add significant value, the field is rapidly moving and we envision that the guidance for best practices will soon be needed. We hope the current perspective and review provides a snapshot of the rapidly developing field and evolves into such guidance with the continuous contribution of the QSP + ML community.

## Glossary

Black box: A model for which the inputs and outputs are observable but not the internal workings e.g. it is not possible to determine which features of an image are used by facial recognition algorithms
Constraint-based approaches: Approaches to generate a solution to a mathematical model that impose conditions such as minimum and maximum values
Deep neural networks: Neural network inputs and outputs are connected via one or more hidden layers. Deep neural networks are those with many hidden layers that allow the neural network to learn more complex patterns in training data
Digital biomarkers: Physiological and behavioral data collected and measured by means of digital devices such as a Holter monitor
Feature selection: The process of selecting a subset of data to aid in interpretation
Generative adversarial network: A field of ML that uses an unsupervised “game” to improve a neural network model. The generator generates neural networks and an adversarial network compares outputs: both improve over repeated cycles
Grey box: A model that combines known and estimated (unknown) terms and/or equations
Machine learning (ML): The study of computer algorithms that learn and adapt without following explicit instructions, to analyze and draw inferences from patterns in data
Neural network (NN): A mathematical function built from layers of nonlinear transformations. The approach is inspired by the way neurons are hypothesized to interact during learning: a network connection strengthens if is excitatory and weakens if inhibitory.
Pharmaco-pathomics: Automated machine learning-based classification of pathology images generated in clinical pharmacology trials
Predictive Modeling: Modeling that predicts the response of a change to the system
QSP modelling: Modeling to describe quantitative interactions between a drug and the human system
Rich mutliplexed data: Multiple datasets generated from a single set of samples. Implies profiling data such as transcriptomics, proteomics, and metabolomics
Scientific machine learning: A form of machine learning which incorporates mechanistic scientific laws into the learning process or architectures [94]
Surrogate machine learning models: Machine learning models trained to emulate the input output behavior of scientific simulations. Usually, these models are trained as accelerated oracles for computing how simulations will perform at new parameters.
White box: A model with known, observable and/or interpretable relationships between variables.
